# miRNA-200c inhibits invasion and metastasis of human non-small cell lung cancer by directly targeting ubiquitin specific peptidase 25

**DOI:** 10.1186/1476-4598-13-166

**Published:** 2014-07-06

**Authors:** Jing Li, Qiang Tan, Mingxia Yan, Lei Liu, Hechun Lin, Fangyu Zhao, Guoliang Bao, Hanwei Kong, Chao Ge, Fanglin Zhang, Tao Yu, Jinjun Li, Xianghuo He, Ming Yao

**Affiliations:** 1 State Key Laboratory of Oncogenes and Related Genes, Shanghai Cancer Institute, Renji Hospital, Shanghai Jiao Tong University School of Medicine, 200032 Shanghai, China; 2 Department of Shanghai Lung Tumor Clinic Center, Shanghai Chest Hospital, School of Medicine, Shanghai Jiao Tong University, 200030 Shanghai, China

**Keywords:** miR-200c, Metastasis, NSCLC, Ubiquitin specific peptidase 25

## Abstract

**Background:**

Growing evidence indicates that miR-200c is involved in carcinogenesis and tumor progression in non-small-cell lung cancer (NSCLC). However, its precise biological role remains largely elusive.

**Methods:**

The functions of miR-200c and USP25 in migration/invasion and lung metastasis formation were determined by transwell and tail vein injection assays, respectively. The potential regulatory targets of miR-200c were determined by prediction tools, correlation with target protein expression, and luciferase reporter assay. The mRNA expression levels of miR-200c and USP25 were examined in NSCLC cell lines and patient specimens using quantitative reverse transcription-PCR. The protein expression levels of USP25 were examined in NSCLC cell lines and patient specimens using western blot and immunohistochemical staining.

**Results:**

We demonstrated that over-expression of miR-200c inhibited NSCLC cells migration, invasion, epithelial-mesenchymal transition (EMT) in vitro and lung metastasis formation in vivo. Further studies revealed that USP25 was a downstream target of miR-200c in NSCLC cells as miR-200c bound directly to the 3’-untranslated region of USP25, thus reducing both the messenger RNA and protein levels of USP25. Silencing of the USP25 gene recapitulated the effects of miR-200c over-expression. Clinical analysis indicated that miR-200c was negatively correlated with clinical stage, lymph node metastasis in NSCLC patients. Moreover, USP25 protein and mRNA level expressions were higher in NSCLC patients, compared to healthy control, and correlated with clinical stage and lymphatic node metastasis.

**Conclusions:**

These findings indicate that miR-200c exerts tumor-suppressive effects for NSCLC through the suppression of USP25 expression and suggests a new therapeutic application of miR-200c in the treatment of NSCLC.

## Background

The leading cause of cancer mortality is lung cancer in the worldwide. Non–small cell lung cancer (NSCLC) is the most common type of lung cancer, accounting for more than 85% of all lung cancer cases [1]. Despite the enormous improvements made in chemotherapy and radiotherapy over the past few decades, the outlook for patients with NSCLC was dismal, with only slightly more than 15% of patients alive 5 years after diagnosis. NSCLC can be further classified into adenocarcinomas, carcinoma, large cell carcinoma and bronchoalveolar carcinomas (BAC) [2]. The distant metastases are responsible for the failure of lung cancer therapy and the poor prognosis of lung cancer. However, the mechanisms of metastasis have not yet been fully elucidated.

MicroRNAs (miRNAs) are small, non-coding RNA molecules that negatively regulate gene expression, mainly through direct interaction with the 3’-untranslated region (3’-UTR) of corresponding target messenger RNAs (mRNA) [3]. After binding to target mRNAs, miRNAs form a complex with target mRNAs and decrease the levels of the encoded protein, either by degrading the mRNA or by suppressing translation of the target mRNA [4]. It has been reported that miRNAs can post-transcriptionally regulate 30% of human genes, thereby suggesting that miRNAs may have pivotal roles in physiological and pathological processes, including human carcinogenesis [5]. Over the past 10 years, evidence has emerged that miRNAs were crucial for the initiation, promotion, and progression of human cancers. A large number of miRNAs have recently been implicated in cancer metastasis [6]. For example, miR-155, miR-222, miR-210, miR-107, and miR-10a have a role in pancreatic cancer [7-9], miR-148a, miR-23a, and miR-193a in hepatocellular carcinoma [10-12], and miR-200, miR-218 in gastric cancer [13,14]. Furthermore, miR-133b, miR-143 and miR-32 in colorectal cancer [15-17]. However, only a few miRNAs known involved in NSCLC metastasis have been reported. A better understanding of the changes in miRNA expression during NSCLC invasion may lead to a better understanding of NSCLC development, as well as to possible improvements in the diagnosis and treatment of advanced NSCLC.

Previously, we established a highly invasive (SPC-A-1sci) cell subline and a weakly invasive (SPC-A-1) cell subline by in vivo selection in NOD/SCID mice [18]. We compared the global miRNA profiles of SPC-A-1sci and SPC-A-1 cell, and revealed low expression levels of miR-200c had influence on the invasion and migration ability of NSCLC cell lines [19]. In this work, miR-200c has been investigated in much greater detail, because miR-200c has been reported to be correlated with EMT [20], and SPC-A-1sci cells display phenotypic changes consistent with EMT [18]. Recently, miR-200c has been reported in several tumors, including breast cancer, lung cancer, esophageal cancers, colorectal cancer, and pancreatic cancer [21-25]. These findings indicate that miR-200c may function importantly in human carcinogenesis. However, for miR-200c, the potential roles and related target genes in NSCLC metastasis are still not well elucidated.

## Results

### MiR-200c suppresses the migration and invasion of NSCLC cells in vitro

**Figure 1:**
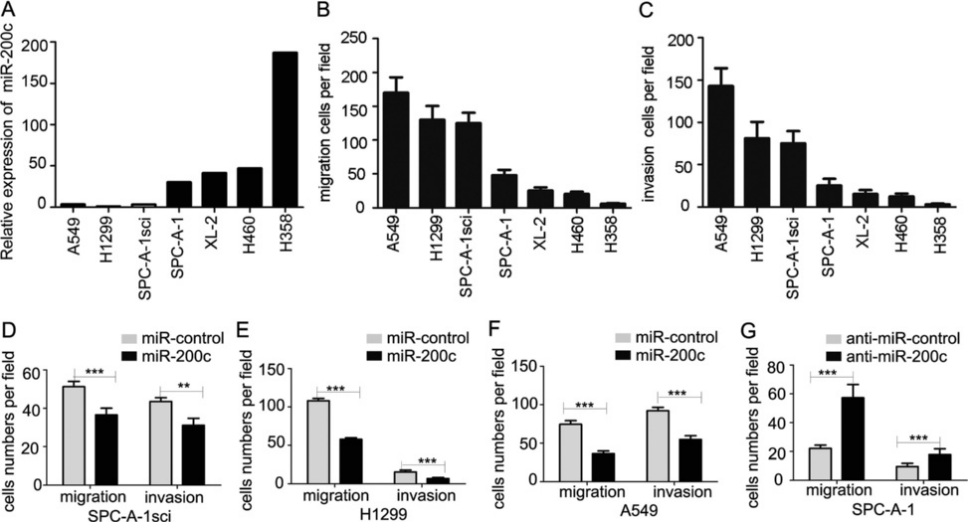
**Correlation analysis of miR-200c expression and invasion capacity. (A)** Levels of miR-200c expression in 7 human NSCLC cell lines were measured by quantitative real-time RT-PCR. U6 was used for normalization. **(B and C)** The invasion and migration ability of 7 human NSCLC lines was determined by using the matrigel migration and invasion assay. **(D-G)** Transwell migration and invasion assays for SPC-A-1sci, H1299, and A549 cells were performed after transduction with by miR-200c mimics or miR-controls, and SPC-A-1 cells were performed after transduction with miR-200c inhibitor or miR-control. The results are representative of at least three independent experiments. Statistical analysis was performed using Student’s *t-*test. Error bars represent S.E.M. *P < 0.05; **P < 0.01; ***P < 0.001.

To clarify the significance of miR-200c in human NSCLC metastasis, we investigated the expression of miR-200c in7 human NSCLC cell lines by qRT-PCR. The expression levels of miR-200c were higher in the SPC-A-1, XL-2, H460, and H358 cell lines than in the A549, H1299, and SPC-A-1sci cell lines (Figure 1A). The migration and invasion ability of 7 human NSCLC cell lines were compared by using transwell assays with or without matrigel. All cell lines higher-expressing miR-200c (SPC-A-1, XL-2, H460, and H358) showed fewer cells than the cell lines with low levels of miR-200c (A549, H1299, and SPC-A-1sci) (Figure 1B,C).

On the basis of the results showing the suppression of miR-200c expression in high metastatic NSCLC cells, loss- and gain-of-function studies of miR-200c were conducted using a transient transfection strategy with miR-200c mimics or inhibitors (Additional file 1: Figure S1A and B). The invasion and migration ability of A549, H1299 and SPC-A-1sci cells transfected with miR-200c mimics were significantly decreased when compared with the invasion and migration ability of the control cells (Figure 1D-F). On the other hand, silencing of miR-200c by transfecting the miR-200c inhibitor into the SPC-A-1 cells increased cell invasion and migration ability (Figure 1G). Therefore, the expression of miR-200c may affect the invasion and migration of human NSCLC cells in vitro.

### Over-expression of miR-200c impairs the formation of metastases of NSCLC cells in vivo

**Figure 2:**
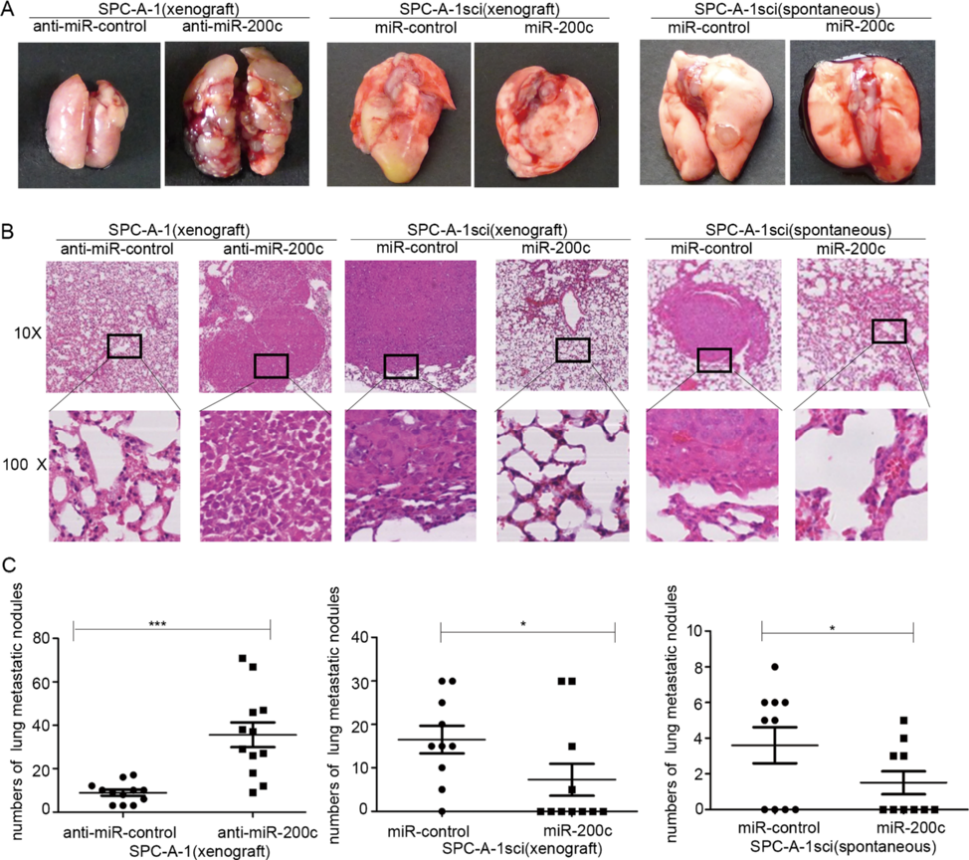
**Overexpression of miR-200c suppress NSCLC cells metastases in vivo. (A and B)** Representative gross photo of a mouse lung and the images of histological inspection of a mouse lung for the presence of microscopic lesions twelve weeks after tail vein injection (xenograft) with SPC-A-1 cells stably expressing the miR-200c inhibitor or miR-control inhibitor lentiviral vector, and eight weeks after tail vein injection (xenograft), twelve weeks after subcutaneous injection (subcutaneous) with SPC-A-1sci cells stably expressing the miR-200c or miR-control lentiviral vector. **(C)** Quantification of lung microscopic nodules in the lungs of each group. Statistical analysis was performed using Student’s *t-*test. Error bars represent S.E.M. *P < 0.05; **P < 0.01; ***P < 0.001.

We asked whether expression of miR-200c would also affect metastatic behaviors in vivo. To address this possibility, SPC-A-1 transfected with miR-200c inhibitor or miR-control inhibitor lentiviral vector, SPC-A-1sci with miR-200c or miR-control lentiviral vector were inoculated into nude mice through the lateral tail vein (Additional file 2: Figure S2). After twelve weeks, lung metastasis was apparent in mice injected SPC-A-1 transfected with miR-200c inhibitor lentiviral vector compared with negative control cells. In stark contast, few metastatic tumors were detected in mice injected SPC-A-1sci with miR-200c lentiviral vector compared with control group for eight weeks (Figure 2A). Similar trends are observable in histologic analysis (Figure 2B). The numbers of lung metastasis nodules were significantly increased in miR-200c inhibitor lentiviral vector group, decreased in miR-200c lentiviral vector group, respectively, when compared with control group (Figure 2C).Moreover, SPC-A-1sci cells stably expressing miR-control or miR-200c lentiviral vector were injected subcutaneously into the right upper flank region of five-week-old NOD/SCID mice. After twelve weeks, the results showed that increased expression of miR-200c impaired NSCLC cells the formation of metastases (Figure 2). These results were similar through the lateral tail vein. Taken together, our results suggest that miR-200c is a negative regulator for NSCLC metastasis.

### MiR-200c expression modulates the epithelial-mesenchymal transition (EMT)

**Figure 3:**
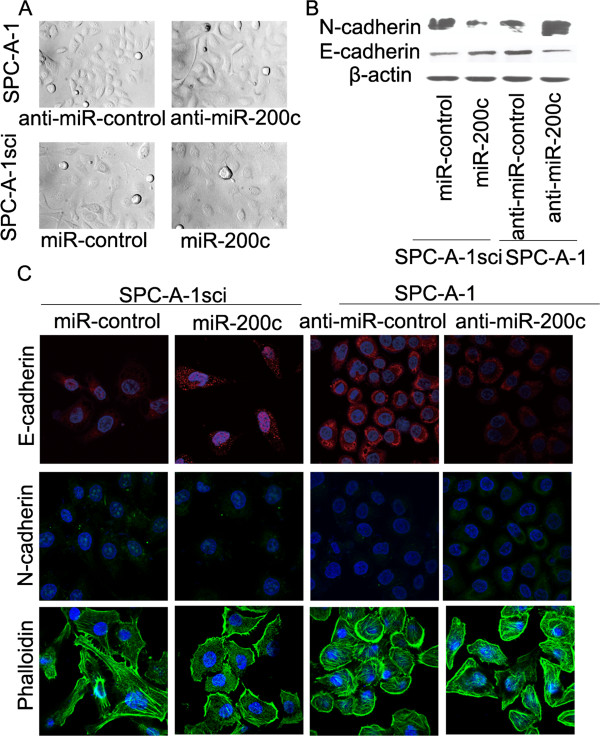
**miR-200c regulated the epithelial-mesenchymal transition (EMT). (A)** SPC-A-1 cells were transfected with miR-control inhibitor or miR-200c inhibitor lentiviral vector, and SPC-A-1sci cells were transfected with the miR-control or miR-200c lentiviral vector. Light microscope pictures were taken after transfection (100x magnification). **(B)** The protein levels of E-cadherin (epithelial marker) and N-cadherin (mesenchymal marker) were determined by western blot analyses after transfection with miR-control inhibitor or miR-200c inhibitor lentiviral vector, and SPC-A-1sci cells were transfected with the miR-control or miR-200c lentiviral vector. β-actin served as an internal control. **(C)** Immunofluorescence staining of E-cadherin, N-cadherin, and Phalloidin, after transfection with miR-control inhibitor or miR-200c inhibitor lentiviral vector, and SPC-A-1sci cells were transfected with the miR-control or miR-200c lentiviral vector. The red, green signal represents the staining of the indicated proteins, and the blue signal represents the nuclear DNA staining by DAPI. Magnification, 600×. The results are representative of at least three independent experiments.

A typical morphological change of SPC-A-1sci, SPC-A-1 was noted after treatment with miR-200c lentiviral vector or miR-200c inhibitor lentiviral vector. Compared with the control cells, the results showed that a significant decrease in spindle-shaped cells was paralleled by an increase in rounded cells in miR-200c-transduced SPC-A-1sci cells. In contrast, more rounded cells became spindle-shaped in SPC-A-1 cells transfected with miR-200c inhibitor lentiviral vector (Figure 3A). E-cadherin and N-cadherin protein expression levels were measured by Western blotting. Up-regulated miR-200c expression levels enhanced E-cadherin protein expression, but inhibited N-cadherin protein expression, and that down-regulated miR-200c expression levels inhibited E-cadherin protein expression, but enhanced N-cadherin protein expression (Figure 3B). In addition, we examined the E-cadherin, N-cadherin, Phalloidin expression by confocal immunofluorescence. Similar results were also found in immunofluorescence, whereas the changes of E-cadherin and N-cadherin by immunostaining were not as sharp as that observed by western blot. E-cadherin and N-cadherin were increased and decreased, respectively, in miR-200c-transduced SPC-A-1sci cells. In contrast, E-cadherin and N-cadherin were decreased and increased, respectively, in miR-200c-inhibitor-treated SPC-A-1 cells (Figure 3C). These observations reveal that miR-200c can inhibit the EMT of NSCLC cells.

Evidence has emerged that miR-200c directly targets one of the major EMT transcription factor ZEB1 and in turn suppresses EMT. We analyzed the expression of ZEB1 by qRT-PCR and western blotting, the results showed that a corresponding reduction in mRNA and protein levels were also detectable for ZEB1 in response to miR-200c up-regulation in SPC-A-1sci cells. Conversely, increased mRNA and protein expressions for ZEB1 were also demonstrated following transfection with the miR-200c inhibitor in SPC-A-1 cells (Additional file 3: Figure S3A and B). We next analyzed the expressions of E-cadherin, and N-cadherin, when the expression of ZEB1 was knocked down by siRNA in SPC-A-1sci cells. The results showed ZEB1 suppressed EMT in NSCLC (Additional file 3: Figure S3C). Taken together, these results showed that miR-200c could suppress EMT by directly targeting ZEB1. The results were consistent with other reports.

### USP25 is a direct target of miR-200c during NSCLC metastasis

**Figure 4:**
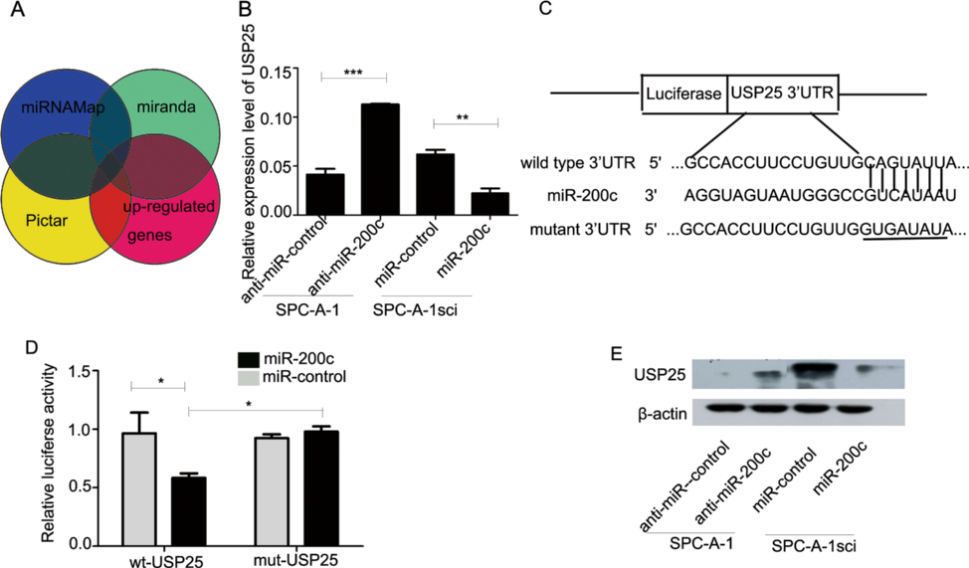
**USP25 expression is downregulated by miR-200c directly targeting of the 3’-UTR of USP25. (A)** USP25 was found for the potential regulatory targets of miR-200c by considering the up-regulated genes from the gene chip and using prediction tools, including miRNAMap, miRanda, and PicTar. **(B)** The USP25 mRNA levels were determined by real-time PCR analysis after transfection with the miR-200c mimics or negative control in SPC-A-1sci cells or after transfection with the miR-200c inhibitor or negative control in SPC-A-1cells. β-actin served as an internal control. **(C)** The putative miR-200c binding site in the USP25 3’-UTR. **(D)** Luciferase activity assays for luciferase reporters with wild-type or mutant USP25 3’-UTR were performed after co-transfected with miR-200c mimics or miR-control in 293T cells. The luciferase activity of each sample was normalized to Renilla luciferase activity. **(E)** USP25 protein levels were determined by western blot analyses after transfection with the miR-200c mimics or negative control in SPC-A-1sci cells or after transfection with the miR-200c inhibitor or negative control in SPC-A-1 cells. β-actin served as an internal control. The results are representative of at least three independent experiments. Statistical analysis was performed using Student’s *t-*test. Error bars represent S.E.M. *P < 0.05; **P < 0.01; ***P < 0.001.

To determine how the low level of miR-200c expression contributes to the invasion and metastasis of NSCLC cells, we examined the global mRNA expression profile of SPC-A-1sci and SPC-A-1 cells. We searched for the potential regulatory targets of miR-200c by considering the up-regulated genes from the gene chip and using prediction tools, including miRNAMap, miRanda, and PicTar (Figure 4A). Five potential target genes were selected for validation and further analysis: USP25, SMURF2, ID2, PLK2, PKIA. The down-regulation of only three of these genes: USP25, PKIA, and SMURF2, following ectopic miR-200c up-regulation in SPC-A-1sci, could be confirmed by real-time PCR analysis (Figure 4B and Additional file 4: Figure S4A).

To determine if miR-200c directly targets the 3’UTRs of USP25, PKIA, and SMURF2, we constructed vectors containing the full-length wild-type or mutant 3’-UTR of USP25, PKIA, SMURF2 directly fused to the downstream of the firefly luciferase gene (Figure 4C and Additional file 4: Figure S4B). The wild-type or mutant vector was co-transfected into HEK-293T cells with miR-200c mimics or miR-control. The transfection efficiency was normalized by cotransfection with Renilla reporter vector. The results shown miR-200c significantly decreased the relative luciferase activity of wild-type USP25 and SMURF2 3’-UTR, whereas the reduction of the luciferase activity with mutant USP25, SMURF2 of 3’-UTR was not as sharp as that observed in the wild-type counterpart. The reduction of the luciferase activity with wild-type or mutant PKIA of 3’-UTR was not observed (Figure 4D and Additional file 4: Figure S4C, D). A corresponding reduction at the protein levels was also detectable by Western blot for USP25 and SMURF2 in response to miR-200c up-regulation. Conversely, increased mRNA and protein expressions for USP25 and SMURF2 were also demonstrated following transfection with the miR-200c inhibitor in SPC-A-1 cells (Figure 4E and Additional file 4: Figure S4E).

Next, we investigated which, if any, miR-200c direct targeted mediate the capacity for cellular invasion. USP25 and SMURF2 seemed appealing: USP25 is involved in negative regulation of IL-17-mediated signaling and inflammation by interacting with TRAF5 and TRAF6, and regulates TLR4-dependent innate immune responses through deubiquitination of the adaptor protein TRAF3 [26,27]. But few studies have been performed to determine the significance of USP25 in tumor metastasis; SMURF2 is known to suppress tumor angiogenesis and metastasis [28], However, others reported that SMURF2 promoted metastasis in breast cancer cells [29]. Given their described roles, USP25 and SMURF2 seemed plausible candidates to shape the miR-200c-mediated invasion and migration of NSCLC cells. To determine whether any of them were critical mediators of miR-200c’s role in cellular invasion, we silenced SMURF2 using RNA interference (RNAi) in SPC-A-1sci cell lines (Additional file 5: Figure S5A, B). The results showed that downregulating SMURF2 enhanced invasion (Additional file 5: Figure S5C, D).

### USP25 has a critical role in miR-200c-mediated invasion and migration of NSCLC cells

**Figure 5:**
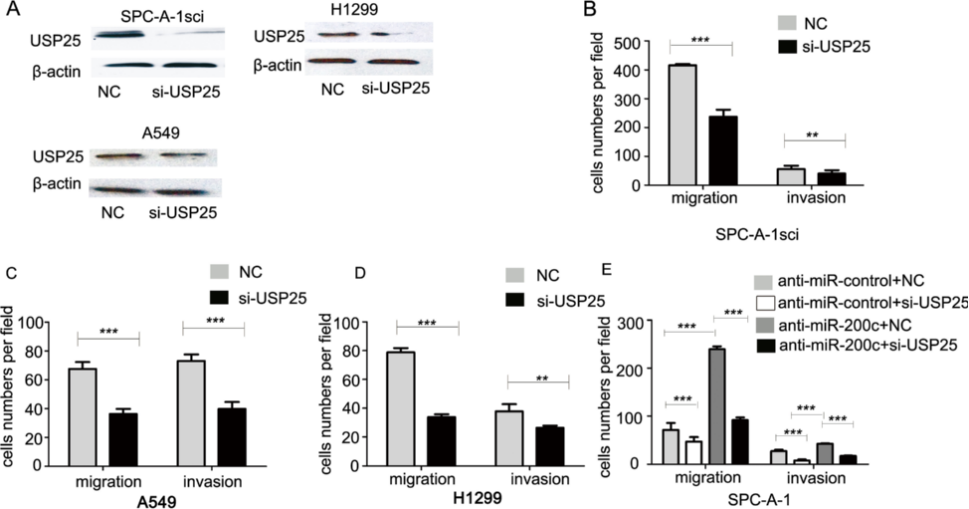
**USP25 functions as a metastasis promoter and its molecular mechanism. (A)** The protein levels of USP25 were determined by western blot analyses after transfection with si-USP25 or a negative control in SPC-A-1sci, A549, H1299 cells, β-actin served as an internal control. **(B-D)** Transwell migration and invasion assays for SPC-A-1sci, A549, and H1299 cells were performed after transfection with si-USP25 or a negative control. **(E)** Transwell migration and invasion assays for SPC-A-1-miR-control and SPC-A-1-anti-miR-200c cells were performed after transduction with si-USP25 or a negative control. The results are representative of at least three independent experiments. Statistical analysis was performed using Student’s *t-*test. Error bars represent S.E.M. *P < 0.05; **P < 0.01; ***P < 0.001.

To determine whether USP25 was critical mediators of miR-200c’s role in cellular invasion, we confirmed that the knock-down of USP25 expression levels by siRNA in A549, H1299 and SPC-A-1sci cells, siRNA remarkably reduced the expression of USP25 levels protein (Figure 5A). Knockdown of USP25 by siRNA in A549, H1299, and SPC-A-1sci cells inhibited cell migration and invasion in vitro, which fell to levels similar to those observed after transfection with the miR-200c mimics (Figure 5B-D). To demonstrate the contribution of USP25 to the biological function of miR-200c, we examined whether the co-transfection of si-USP25 had an effect on miR-200c-inhibitor-induced cell migration and invasion in SPC-A-1 cells (Figure 5E).

These observations suggest that miR-200c directly suppresses USP25-mediated cell invasion. Moreover, over-expression of miR-200c in USP25 knockdown SPC-A-1sci cells were conducted using a transient transfection strategy with miR-200c mimics (Additional file 6: Figure S6A). The invasion and migration ability of USP25 knockdown SPC-A-1sci cells transfected with miR-200c mimics were significantly decreased when compared with the invasion and migration ability of the control cells (Additional file 6: Figure S6B).

### Decreased expression of USP25 inhibits human NSCLC cell metastasis in vivo

**Figure 6:**
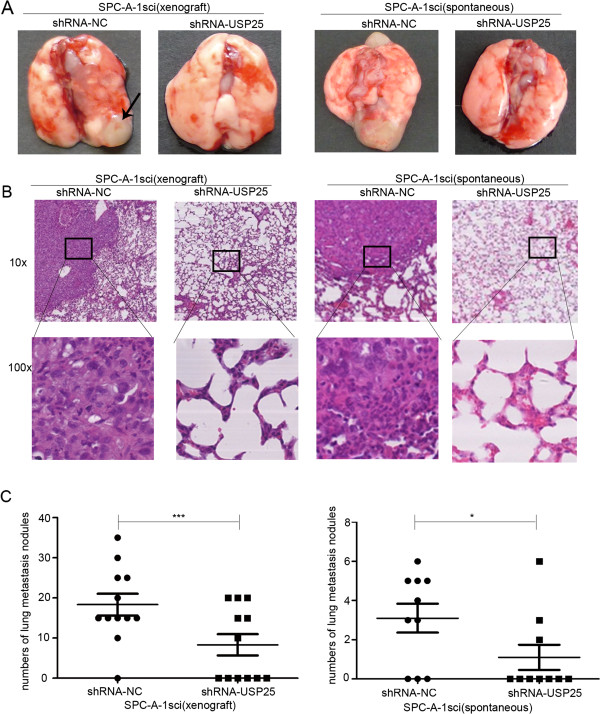
**The promoter roles of USP25 on lung metastasis in vivo. (A, B)** Representative gross photo of a mouse lung and the images of histological inspection of a mouse lung for the presence of microscopic lesions eight weeks after tail vein injection (xenograft) or subcutaneous injection (subcutaneous) with SPC-A-1sci cells stably expressing the sh-USP25 or sh-NC. **(C)** Quantification of lung microscopic nodules in the lungs of each group. Statistical analysis was performed using Student’s *t-*test. Error bars represent S.E.M. *P < 0.05; **P < 0.01; ***P < 0.001.

To further explore the role of USP25 on tumor metastasis in vivo,SPC-A-1sci cells with stably knocked-down USP25 expression (sh-USP25) and the negative control (sh-NC) were inoculated into the tail veins of nude mice or into the right upper flank region of NOD/SCID mice. Lung metastasis was apparent in mice injected with SPC-A-1sci-sh-NC cells. In contrast, few metastatic tumors were detected in mice injected with SPC-A-1sci-sh-USP25 cells. The histological examination of lung tissue indicated that the number of lung metastatic nodules significantly decreased in mice inoculated with shRNA-USP25 cells compared to shRNA-NC cells (Figure 6). In short, our results indicate that decreased expression of USP25 inhibits human NSCLC cell metastasis in vivo.

### miR-200c, and USP25 expression in NSCLC

**Figure 7:**
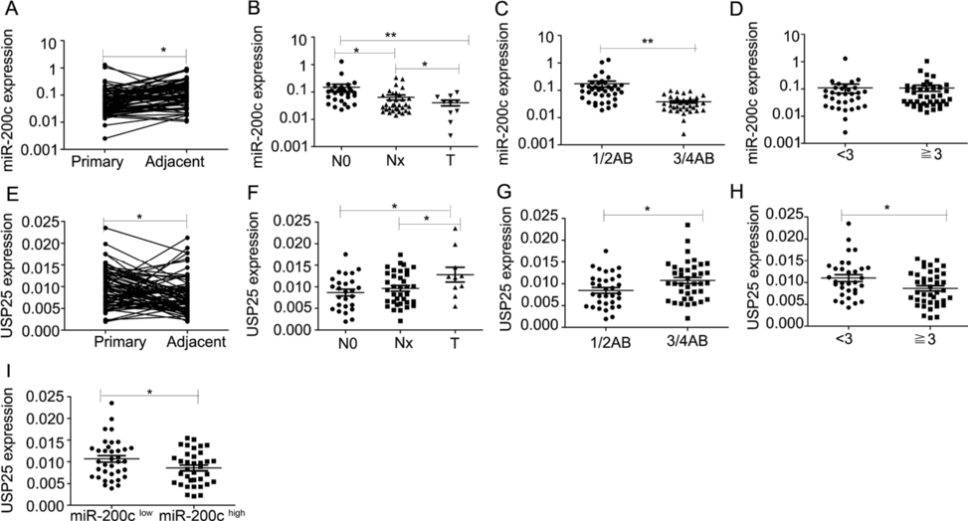
**Clinical validation of miR-200c and USP25. (A,E)** Real-time PCR analysis to quantify the levels of miR-200c, USP25 in NSCLC tissues and non-tumor tissues. **(B,F)** Real-time PCR analysis to quantify the levels of miR-200c, USP25 in NSCLC tissues with lymphatic metastasis (Nx), distant metastasis (T) or without metastasis (N0). **(C,G)** Real-time PCR analysis to quantify the levels of miR-200c, USP25 in the NSCLC patients with clinical early-stage (1/2AB) or advanced-stage (3/4AB). **(D,H)** Real-time PCR analysis to quantify the levels of miR-200c, USP25 in the NSCLC patients with tumor size <3 cm or >3 cm. **(I)** Real-time PCR analysis the levels of USP25 in patients with low and high miR-200c expression levels. Statistical analysis was performed using Student’s *t-*test. Error bars represent S.E.M. *P < 0.05; **P < 0.01; ***P < 0.001.

Based on the similar results of experiments using NSCLC cell lines and xenograft models, it appears that the decreased expression of miR-200c would promote NSCLC invasion and metastasis through USP25. To determine the potential clinicopathological implications of altered miR-200c expression, we investigated the expression levels of miR-200c in 73 NSCLC tissues and non-tumor tissues by qRT–PCR. The results verified that the miR-200c expression level in NSCLC tissues was lower than that in non-tumor tissues (Figure 7A). Statistically significant associations between the miR-200c expression level and clinical stage and between the miR-200c expression level and NSCLC metastasis were observed in this study. The expression of miR-200c was lower in NSCLC patients with lymphatic metastasis, distant metastasis than in those without lymphatic metastasis (Figure 7B). Further analysis showed that miR-200c levels were significantly lower in the NSCLC patients with clinical advanced stage (3/4AB) than early-stage (1/2AB) (Figure 7C), but did not correlate with tumor size (Figure 7D). These data supported decreased miR-200c expression in NSCLC was associated with advanced clinical stage, lymph node metastasis.Next, we investigated the expression levels of USP25 in these patients, the results revealed higher mRNA levels of USP25 were found in NSCLC tissues than non-tumor tissues (Figure 7E), correlated with distant metastasis, and clinical advanced stage (Figure 7F,G). In addition, the level of USP25 had a significant inverse correlation with tumor size (Figure 7H). Lastly, we investigated whether miR-200c level was associated with the mRNA levels of USP25 in these patients. The result revealed tumors with a low level of miR-200c tended to express high levels of USP25, whereas, tumors with a high level of miR-200c tended to express low levels of USP25 (Figure 7I). Hence, it appears that miR-200c negatively regulates tumor metastasis in NSCLC patients by targeting USP25.

### USP25 protein levels in NSCLC are associated with advanced clinical stage, histological grade, and lymph node metastasis

**Figure 8:**
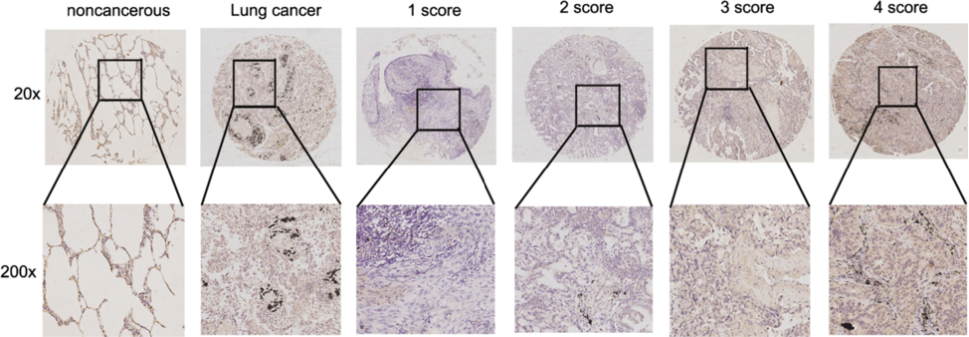
**Representative IHC images of the expression of USP25 between lung cancer and adjacent noncancerous tissues.** Scoring was measured by the percentage of positive cells with the following staining intensities: less than 5% scored “0”; 5–24% scored “1”; 25–49% scored “2”; 50–74% scored “3”; and more than 74% scored “4”.

To further examine the clinical relevance of this finding, we detected USP25 protein levels expression in human NSCLC tissues (n = 256) and matched adjacent normal tissues by Immunohistochemical staining. Correlations between the USP25 expression level and clinicopathologic characteristics of NSCLC are summarized in Table 1 and Figure 8. The high USP25 level correlated positively with poor tumor differentiation (P = 0.0002). The expression of USP25 was higher in the NSCLC patients with clinical advanced stage than early-stage (P = 0.0062). Further analysis showed that USP25 levels were significantly higher in NSCLC patients with lymphatic metastasis than in those without lymphatic metastasis (P < 0.0001). In addition, USP25 levels were higher in primary cancers than in the adjacent controls (P < 0.0001), whereas USP25 levels were negatively related to the tumor size. With respect to clinicopathologic features, we found that high protein levels of USP25 positively correlated with clinical stage, histological grade, and lymphatic metastasis in NSCLC patients, indicating that USP25 plays an important role in NSCLC progression.

**Table 1:** Correlation between secreted USP25 levels in NSCLC patients and their clinicopathologic characteristics

**Clinical pathology**	**USP25 levels (IHC)**			**N**	**P value**
	**–+**	**+ +**	**+ + +-+ + + +**		
Gender					
Male	54(29.2)	49(26.5)	82(44.3)	185	0.0066*
Female	10(14.1)	18(25.4)	43(60.5)	71	
Age					
≦63	30(21.9)	34(24.8)	73(53.3)	137	0.0985
>63	34(28.6)	34(28.6)	51(42.8)	119	
Tumor size (cm)					
<3.5	19(20.6)	16(17.4)	57(62)	92	0.012*
3.5-6	25(25)	32(32)	43(43)	100	
>6	20(31.3)	19(29.7)	25(39)	64	
Histological grade					
Ι/Ι-Π	15(36.6)	13(31.7)	13(31.7)	41	0.0002*
Π	33(27)	38(31.1)	51(41.9)	122	
Π-Ш/Ш	16(17.2)	16(17.2)	61(65.6)	93	
Clinical Stage					
1AB	26(41.3)	12(19)	25(39.7)	63	0.0062*
2AB	23(20.4)	35(31)	55(48.7)	113	
3AB/4	15(18.7)	20(25)	45(56.3)	80	
Lymph node status					
Metastasis	24(15.4)	42(26.9)	90(57.7)	156	<0.0001*
No metastasis	40(40)	25(25)	35(35)	100	
Carcinoma					
Primary	64(25)	67(26.2)	125(48.9)	256	<0.0001*
Adjacent	132(51.6)	123(48)	1(0.4)	256	

*P* value represents the probability from a chi-square test for tissue USP25 levels between variable subgroups, *P < 0.05.

## Discussion

In recent years, many studies have shown that the expression of miRNAs is aberrant in human cancer [30]. Identification of tumor-associated miRNAs and their target genes is critical for understanding the roles of miRNAs in tumorigenesis and may be important for novel therapeutic targets [31].

In our previous work, we isolated invasive and non-invasive cell subpopulations from human NSCLC SPC-A-1 cell lines by in vivo selection in NOD/SCID mice [18]. We identified 117 novel metastasis-related miRNAs in NSCLC based on a well-established metastasis cell model [19]. The finding that miR-200c was downregulated in metastatic SPC-A-1sci cells was intriguing, because decreased miR-200c levels have been reported in several other types of tumor [21,23-25], thus indicating that decreased miR-200c may be a common event in the tumorigenesis. Other reports showed serum miR-200c associated with poor prognosis in patients with lung cancer [32]. In H1299 cells miR-200c targets multiple non-small cell lung cancer prognostic markers DLC1, ATRX, and HFE [33]. However, its precise biological role in NSCLC metastasis remains largely elusive.

We focused on the effect of miR-200c on NSCLC metastasis and showed that miR-200c acted as a tumor suppressor during NSCLC metastasis. The expression of miR-200c was negatively correlated with the invasion and migration of NSCLC cell lines in vitro. Moreover, our results suggest that decreased miR-200c levels promoted, increased miR-200c levels inhibited NSCLC cell migration and invasion in vitro and metastasis in vivo. The activity of miR-200c in relation to EMT- associated phenotypes has been extensively studied [34]. In the current study, we also found miR-200c was associated with EMT. Together, these findings suggest that miR-200c functions as a key mediator of metastasis in NSCLC.

As part of our research on how the miR-200c affects NSCLC metastasis, several bioinformatics tools for screening putative miRNA target genes were used, including miRNAMap, PicTar and miRanda and up-regulated genes in gene chip. We demonstrated that USP25 was a critical downstream target of miR-200c. To test this assumption, we investigated whether miR-200c inhibited USP25 mRNA and protein levels, then found that up-regulation of miR-200c led to a significant decrease in USP25 mRNA and protein levels, thereby suggested that USP25 was a functional target of miR-200c. Lastly, the dual-luciferase reporter assays suggested that USP25 was one of the functional downstream targets of miR-200c. The effect of USP25 on tumor metastasis has not been studied. In the current study, we found that knockdown of USP25 expression reduced NSCLC cell metastasis similar to that of the restoration of miR-200c.

To determine the potential clinicopathological implications of altered miR-200c expression, we investigated the expression levels of miR-200c in 73 NSCLC tissues and non-tumor tissues by qRT–PCR. The results showed that the miR-200c expression level in NSCLC tissues was lower than that in non-tumor tissues, and negatively associated with advanced clinical stage, lymph node metastasis. Next, we investigated the expression levels of USP25 in these patients, the results revealed higher mRNA levels of USP25 were found in NSCLC tissues than non-tumor tissues, correlated with distant metastasis, and advancing stage. Lastly, we found a low level of miR-200c tended to express high levels of USP25, whereas tumors with a high level of miR-200c tended to express low levels of USP25. In addition, there was a significant association between USP25 protein levels positively correlated with clinical stage, histological grade, and lymphatic metastasis by Immunohistochemical staining in 256 NSCLC patients. These findings indicated that the invasion suppression effect of miR-200c was at least partly mediated through a decrease in USP25 expression.

USP25 is a member of ubiquitinating-specific proteases (USPs) family, which contain two short, conserved fragments (lysine and histidine boxes) that contain Cys, His and Asn residues, which form a catalytic triad that can remove ubiquitin molecules from large proteins [35]. Ubiquitination is a post-translational protein modification referring to the attachment of the small protein ubiquitin to a lysine residue of a target protein. This modification is more versatile than other post-translational modifications because several ubiquitin molecules can be added to the first, creating chains of ubiquitin on the target proteins that modify the signal [36]. The ubiquitin proteasome system regulates all processes that are involved in carcinogenesis, among them invasion and metastasis, EMT, the process that endows neoplastic cells with invasive and metastatic potential, is intimately interwoven with other neoplastic processes, being served by several common pathways, several of which are regulated by the ubiquitin proteasome system [37]. USP28 is a key regulator of the DNA damage checkpoint, high expression levels of USP28 are found in colon and breast carcinomas, and stabilization of MYC by USP28 is essential for tumour-cell proliferation [38]. USP22 are found significantly associated with progression and unfavorable clinical outcome in esophageal squamous cell carcinoma, NSCLC [39,40]. USP4 is regulated by AKT phosphorylation and directly deubiquitylates TGF-β type I receptor [41].

Recently, USP25 was significantly frequent mutation in HCC [42], and was involved in negative regulation of IL-17-mediated signaling and inflammation by interacting with TRAF5 and TRAF6,and regulates TLR4-dependent innate immune responses through deubiquitination of the adaptor protein TRAF3 [26,27]. Seven members of the TRAF family have been identified (TRAF1-7), and play an important role in a variety of signaling pathways. TRAF6, a unique TRAF family member, possesses a unique receptor-binding specificity, which is important for its crucial role as the signaling mediator for not only the TNF receptor superfamily but also the IL-1R/Toll-like receptor superfamily [43,44]. The downstream signals activated by TRAF6 mainly include NF-κB and AP-1, while NF-κB and AP-1 play an important role in the transcription and expression of numerous genes (including carcinogenesis, invasion and metastasis etc.) in organism [45].

## Conclusions

In this study, we provided first-time evidence that when significantly down-regulated miR-200c promoted NSCLC cell invasion and migration, at least partly through the induction of USP25, which was a potential metastasis promoter in NSCLC. These findings have implications for new mechanisms of miR-200c mediated regulation of lung cancer, and suggest that miR-200c may be a potential therapeutic target for human NSCLC.

## Methods

### Cell culture

The human lung cell lines A549, H1299, SPC-A-1sci, SPC-A-1, XL-2, H460, H358, and HEK-293T were maintained in our laboratory. XL-2 cells were established from the abdomen of a NSCLC patient. All cell lines were routinely maintained in DMEM medium (HyClone) supplemented with 10% fetal bovine serum (Biowest, South America Origin), 100U/ml penicillin sodium, and 100mg/ml streptomycin sulfate at 37°C in a humidified air atmosphere containing 5% CO_2_. Cells were used when they were in the logarithmic growth phase.

### Cell transfections

The miR-200c mimics, and miR-200c inhibitors that were used in this study were synthesized by Ribobio (Guangzhou, China). Genepharma synthesized the USP25 siRNA. The following sequences were as follows: USP25 siRNA sense: 5’-GGGAGUACUUGAAGGUAAATT-3’; anti-sense: 5’-UUUACCUUCAAGUACUCCCTT-3’; negtive control sense: 5’-UUCUCCGAACGUGUCACGUTT-3’; anti-sense: 5’-ACGUGACACGUUCGGAGAATT-3’. Oligonucleotide transfection was performed with Lipofectamine 2000 reagents according to the manufacturer’s instructions (Invitrogen, CA). For migration, invasion, Western blotting assays and real-time quantitative RT-PCR (qRT-PCR), cells were collected 48h after transfection.

### In vitro migration and invasion assays

Cell migration and invasion assays were performed in a 24-well plate with 8-mm pore size chamber inserts (Corning, New York, NY, USA). For migration assays, 5 × 10^4^ cells expressing miR mimic, miR inhibitor, miR control and negative control were placed into the upper chamber per well with the non-coated membrane. For invasion assays, 1 × 10^5^ cells expressing miR mimic, miR inhibitor, miR control and negative control were placed into the upper chamber per well with the Matrigel-coated membrane, which was diluted with serum-free culture medium. In both assays, cells were suspended in 200 ml of DMEM without fetal bovine serum when they were seeded into the upper chamber. In the lower chamber, 800 ml of DMEM supplemented with 10% fetal bovine serum was added.After incubation for some hours at 37°C and 5% CO2, the membrane inserts were removed from the plate, and non-invading cells were removed from the upper surface of the membrane. Cells that moved to the bottom surface of the chamber were fixed with 100% methanol for 20 min and stained with 0.1% crystal violet for 30 min. Then, the cells were imaged and counted in at least 10 random fields using a CKX41 inverted microscope (Olympus,Tokyo, Japan). The assays were conducted three independent times.

### Establishing stable NSCLC cells

The miR-200c lentiviral vector, miR-200c inhibitor lentiviral vector and miR-control lentiviral vector were synthesized by Genepharma. The sh-USP25 lentiviral vector and sh-control lentiviral vector were synthesized by Openbiosystems (USA).

### Real-time quantitative RT-PCR (qRT-PCR)

The expression of miRNAs was measured using the TaqMan stem-loop RT-PCR method from Applied Biosystems (Foster City, CA, USA). Approximately 10 ng of total RNA was reverse-transcribed according to the MicroRNA Reverse Transcription kit and a specific stem-loop primer according to the manufacturer’s instructions (Applied Biosystems, Foster City, CA). Samples were normalized to RNU6B. SYBR green RT-PCR (TaKaRa) was performed to detect USP25. The sequences are as follows: USP25 Sense: 5’-CGGTCCCAAACGATTCCC-3’; Antisense: 5’–CTCCCTGTTCTGTTGTGCT-3’. All RT-PCR experiments were performed on the GeneAmp® PCR System 9700 (Applied Biosystems, Foster City, CA). All RT-PCR assays were carried out using a 7300 Real-Time PCR System with SDS RQ Study software (Applied Biosystems).

### In vivo assays for metastasis

For the experimental metastasis mouse xenograft model, SPC-A-1 cells transfected with miR-control,miR-200c inhibitor lentiviral vector, SPC-A-1sci cells stably expressing miR-control, miR-200c lentiviral vector, knocked-down USP25 expression, the negative control were inoculated into the tail vein of five-week-old BALB/C-nu/nu nude mice(N = 12). For spontaneous metastasis mouse model,SPC-A-1sci cells stably expressing miR-control, miR-200c lentiviral vector, knocked-down USP25 expression, the negative control were injected subcutaneously into the right upper flank region of five-week-old NOD/SCID mice(N = 10). The animals were maintained under specific pathogen free (SPF) conditions. The mice were manipulated and housed according to protocols approved by the Shanghai Medical Experimental Animal Care Commission. Each tumor cell subline was injected 2 × 10^6^ per mouse. After some weeks, the mice were sacrificed, and the lungs were harvested at necropsy and fixed in 10% neutral PB-buffered formalin (pH 7.4). The fixed samples were then embedded in paraffin, and five non-sequential serial sections were obtained per animal. The sections were stained with H&E and analyzed for the presence of metastases.

### Western blot

Cell proteins were extracted and separated in SDS-PAGE gels, and transferred to nitrocellulose filter membranes (Millipore, USA). Western blot analyses were performed according to standard procedures. Western blot loading control was β-actin (Sigma-Aldrich, 1:30000). The following antibodies were used: anti-USP25 (Abcam, 1:1000), anti-E-cadherin (CST, 1:1000), anti-N-cadherin (CST, 1:1000).

### Immunofluorescence and immunohistochemical analysis

Cells were plated and grown on glass slides for 18 ~ 20 hours and fixed with 4% paraformaldehyde. The slides were then blocked and incubated with the following primary antibodies: Anti-E-cadherin was obtained from Santa Cruz (1:25), Anti-N-cadherin was obtained from Santa Cruz (1:25). Finally, the slides were incubated with fluorescence conjugated secondary antibody (Sigma 1:50) and viewed with a Fluoview FV1000 microscope (Olympus, Japan).

### Luciferase assay

Total cDNA from HUVEC cells was used to amplify the 3’UTR of USP25 by PCR, using the forward primer: 5’-CCGCTCGAGACTGCACACTTTCCCTGAACACAC-3' ; the reverse primer:5’–GAATGCGGCCGCAGAATGTCATAAAATATTAAGGATACTTTCTTTAC-3'. Construction of the 3’-UTR of the USP25-mutant by PCR was performed, using the forward primer: 5’–CCTGTTGGTGATATACTTTGCTTTTATCTTTTC-3'; and the reverse primer:5’–CAAAGTATATCACCAACAGGAAGGTGGCAAAAG-3'. The XhoI and NotI restriction enzyme sites were used. HEK293T cells were plated into 96-well plates at 50% confluence 24 h before transfection. The pmiR report-control vector and Luc-USP25, Luc-USP25-mu, or Luc-control vectors were co-transfected into HEK293T cells using Lipofectamine 2000. Lysates were prepared at 48 h post-transfection. Luciferase activity was measured using a Dual-Luciferase Reporter System (Promega).

### Human NSCLC tissues

Patient samples in this study were obtained following informed consent, according to an established protocol approved by the Ethic Committee of Shanghai Jiao-Tong University School. The data did not contain any information that may lead to the identification of the patients. 73 NSCLC tissues and matched adjacent noncancerous tissues for RNA extraction were provided by Shanghai chest hospital. Of these 73 NSCLC tissues, 16 were squamous cell carcinomas and 57 were non squamous cell carcinomas. Matched pairs of NSCLC tissues and matched adjacent noncancerous tissues were used for the construction of a tissue microarray (Shanghai Biochip Co., Ltd. Shanghai, China) as previously described [46]. There were 256 NSCLC tissues and matched adjacent noncancerous tissues in the tissue microarray. Of these 256 NSCLC tissues, 134 were squamous cell carcinomas and 122 were non squamous cell carcinomas. Immunohistochemical staining was performed to detect the expression of USP25 in NSCLC tissues and matched non-cancerous tissues. The primary antibody against USP25 was obtained from Abcam (1:50). Scoring was measured by the percentage of positive cells with the following staining intensities: less than 5% scored “–”; 5–24% scored “+”; 25–49% scored “++”; 50–74% scored “+++”; and more than 74% scored “++++”.

### Statistical analysis

The results are presented as mean ± SD. Comparisons of quantitative data were analyzed by Student’s *t-*test between two groups (two-tailed; *P* < 0.05 was considered significant). Fisher’s exact test was used to compare qualitative variables. Analysis was performed with SAS 9.0 for Windows.

## Competing interests

The authors declare that they have no competing interests.

## Authors’ contributions

Conceived and designed the experiments: MY, XHH, JJL Performed the experiments: JL, QT, MXY, LL, HCL, FYZ, CG, TY, FLZ. Analyzed the data: JL Contributed reagents/materials/analysis tools: GLB, HW, K Wrote the paper: JL. All authors read and approved the final manuscript.

## Supplementary Material

Additional file 1: Figure S1 Real-time PCR demonstrated the relative miRNA levels. **(A)** The miR-200c was determined by real-time PCR analysis after SPC-A-1sci, A549, H1299 cells transfected with miR-control or miR-200c mimics. **(B)** The miR-200c, miR-200a, miR-200b, miR-141, miR-429 were determined by real-time PCR analysis after SPC-A-1 transfected with miR-control inhibitor or miR-200c inhibitor .U6 served as an internal control. Click here for file

Additional file 2: Figure S2 Real-time PCR demonstrated miR-200c levels.The miR-200c was determined by real-time PCR analysis after SPC-A-1 transfected with miR-control inhibitor or miR-200c inhibitor lentiviral vector, and SPC-A-1sci cells were transfected with the miR-control or miR-200c lentiviral vector. U6 served as an internal control. Click here for file

Additional file 3: Figure S3 ZEB1 regulated the epithelial-mesenchymal transition (EMT). **(A,B)** ZEB1 mRNA and protein levels were determined by real-time PCR and western blot analyses after transfection with the miR-200c mimics or negative control in SPC-A-1sci cells or after transfection with the miR-200c inhibitor or negative control in SPC-A-1cells. β-actin served as an internal control. **(C)** E-cadherin, N-cadherin, ZEB1 protein levels were determined by western blot analyses after transfection with si-ZEB1 or a negative control in SPC-A-1sci cells. β-actin served as an internal control. Click here for file

Additional file 4: Figure S4 Other target genes of miR-200c. **(A)** The ID2, SMURF2, PKIA, and PLK2 mRNA levels were determined by real-time PCR analysis after transfection with the miR-200c mimics or negative control in SPC-A-1sci cells or after transfection with the miR-200c inhibitor or negative control in SPC-A-1cells. β-actin served as an internal control. **(B)** The putative miR-200c binding site in the SMURF2, PKIA 3’-UTR. **(C,D)** Luciferase activity assays for luciferase reporters with wild-type or mutant SMURF2, PKIA 3’-UTR were performed after co-transfected with miR-200c mimics or miR-control in 293T cells. The luciferase activity of each sample was normalized to Renilla luciferase activity. **(E)** The SMURF2 protein levels were determined by western blot analyses after transfection with the miR-200c mimics or negative control in SPC-A-1sci cells or after transfection with the miR-200c inhibitor or negative control in SPC-A-1cells. β-actin served as an internal control. Click here for file

Additional file 5: Figure S5 Downregulating SMURF2 enhanced human NSCLC cell invasion in vitro. **(A,B)** SMURF2 mRNA and protein levels were determined by real-time PCR and western blot analyses after transfection with si-SMURF2 or a negative control in SPC-A-1sci cells. β-actin served as an internal control. **(C,D)** Transwell migration and invasion assays for SPC-A-1sci cells were performed (100x magnification) after transfection with si-SMURF2 or a negative control. The results are representative of at least three independent experiments. Statistical analysis was performed using Student’st-test. Error bars represent S.E.M. *P<0.05; **P<0.01; ***P<0.001. Click here for file

Additional file 6: Figure S6 Over-expression of miR-200c in USP25 knockdown SPC-A-1sci cells decreased the invasion and migration ability. **(A)** USP25 protein levels were determined by western blot analyses after over-expression of miR-200c in USP25 knockdown SPC-A-1sci cells. β-actin served as an internal control. **(B)** Transwell migration and invasion assays were performed after over-expression of miR-200c in USP25 knockdown SPC-A-1sci cells. The results are representative of at least three independent experiments. Statistical analysis was performed using Student’st-test. Error bars represent S.E.M. * P<0.05; ** P<0.01; ***P<0.001. Click here for file
