# Supplementary figures and images for: miRNA-200c inhibits invasion and metastasis of human non-small cell lung cancer by directly targeting ubiquitin specific peptidase 25
Source: Mol Cancer. 2014 Jul 6;13:166. doi: 10.1186/1476-4598-13-166 (PMC4105889; doi:10.1186/1476-4598-13-166)

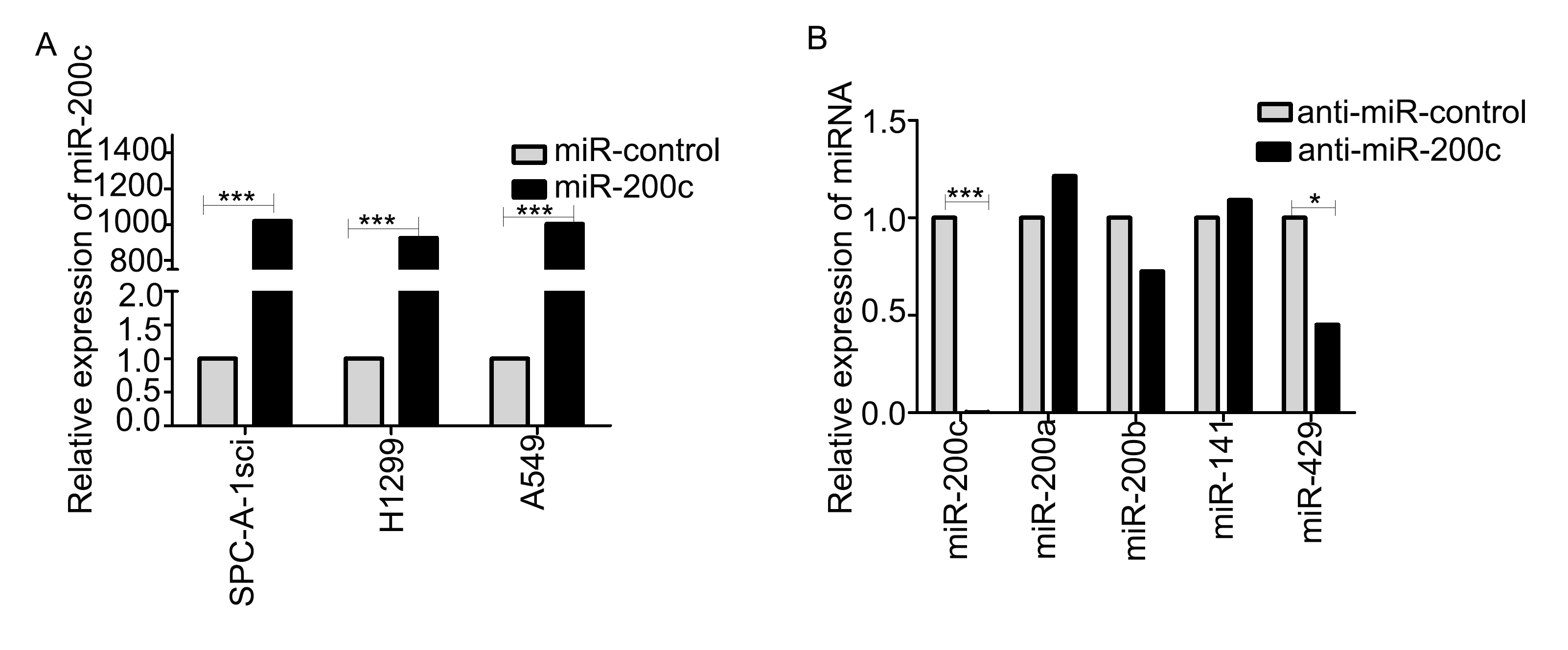

Supplement: Additional file 1: Figure S1 — Real-time PCR demonstrated the relative miRNA levels. (A) The miR-200c was determined by real-time PCR analysis after SPC-A-1sci, A549, H1299 cells transfected with miR-control or miR-200c mimics. (B) The miR-200c, miR-200a, miR-200b, miR-141, miR-429 were determined by real-time PCR analysis after SPC-A-1 transfected with miR-control inhibitor or miR-200c inhibitor .U6 served as an internal control. [file 1476-4598-13-166-S1.tiff]

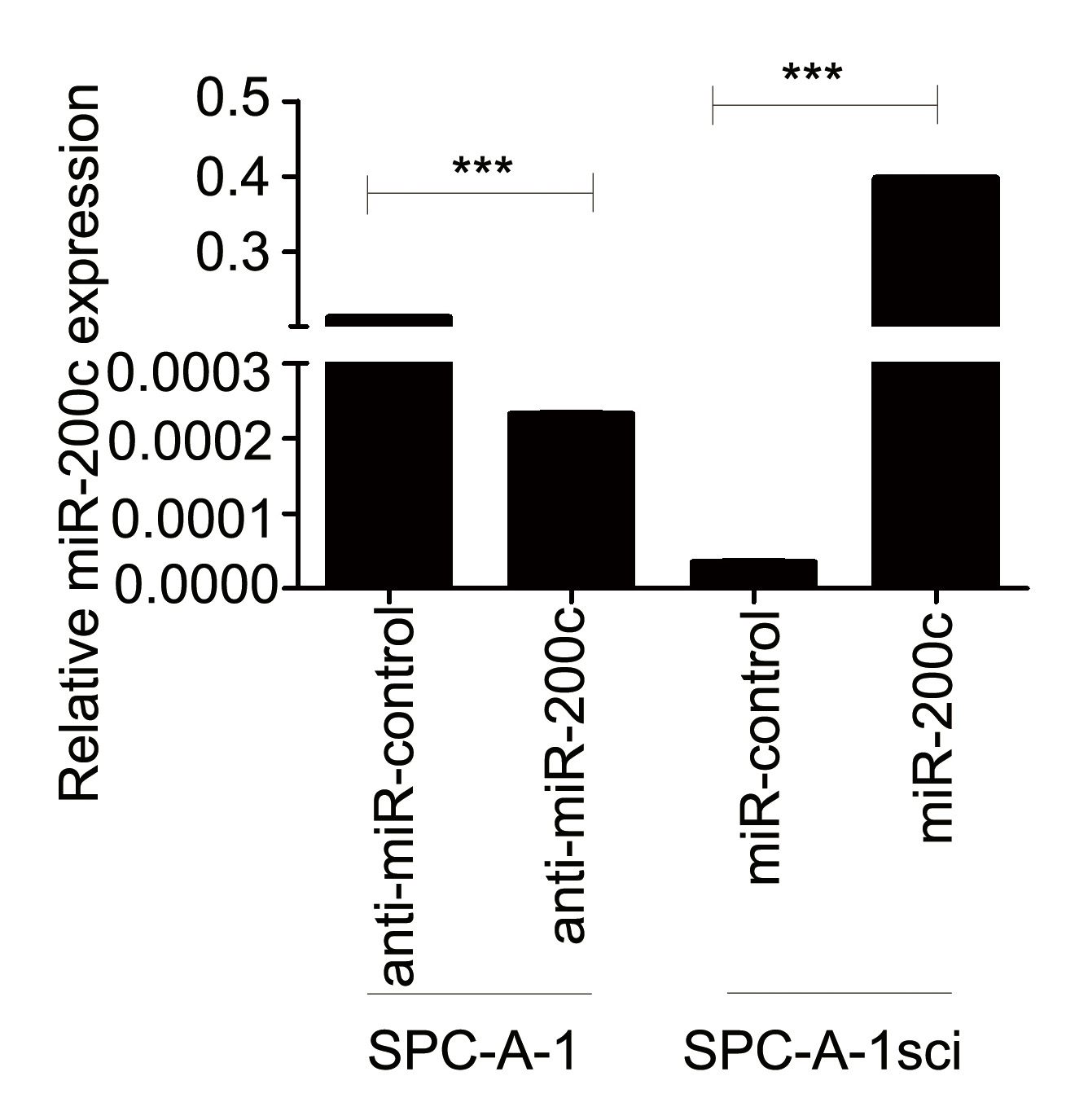

Supplement: Additional file 2: Figure S2 — Real-time PCR demonstrated miR-200c levels.The miR-200c was determined by real-time PCR analysis after SPC-A-1 transfected with miR-control inhibitor or miR-200c inhibitor lentiviral vector, and SPC-A-1sci cells were transfected with the miR-control or miR-200c lentiviral vector. U6 served as an internal control. [file 1476-4598-13-166-S2.tiff]

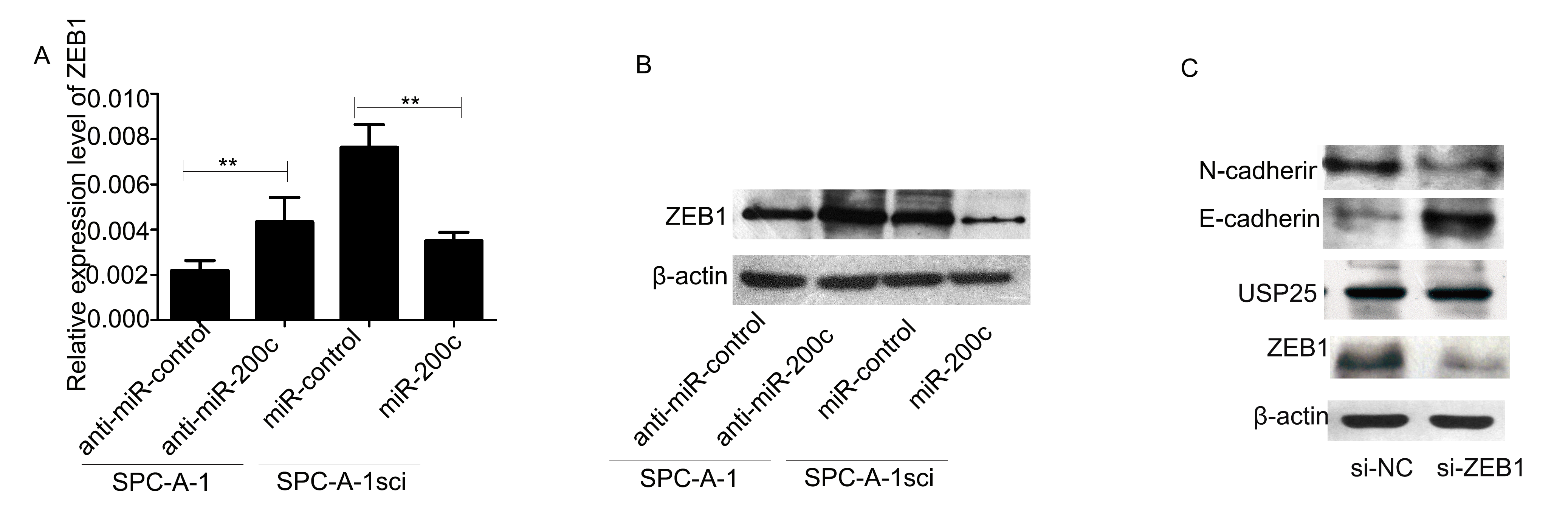

Supplement: Additional file 3: Figure S3 — ZEB1 regulated the epithelial-mesenchymal transition (EMT). (A,B) ZEB1 mRNA and protein levels were determined by real-time PCR and western blot analyses after transfection with the miR-200c mimics or negative control in SPC-A-1sci cells or after transfection with the miR-200c inhibitor or negative control in SPC-A-1cells. β-actin served as an internal control. (C) E-cadherin, N-cadherin, ZEB1 protein levels were determined by western blot analyses after transfection with si-ZEB1 or a negative control in SPC-A-1sci cells. β-actin served as an internal control. [file 1476-4598-13-166-S3.tiff]

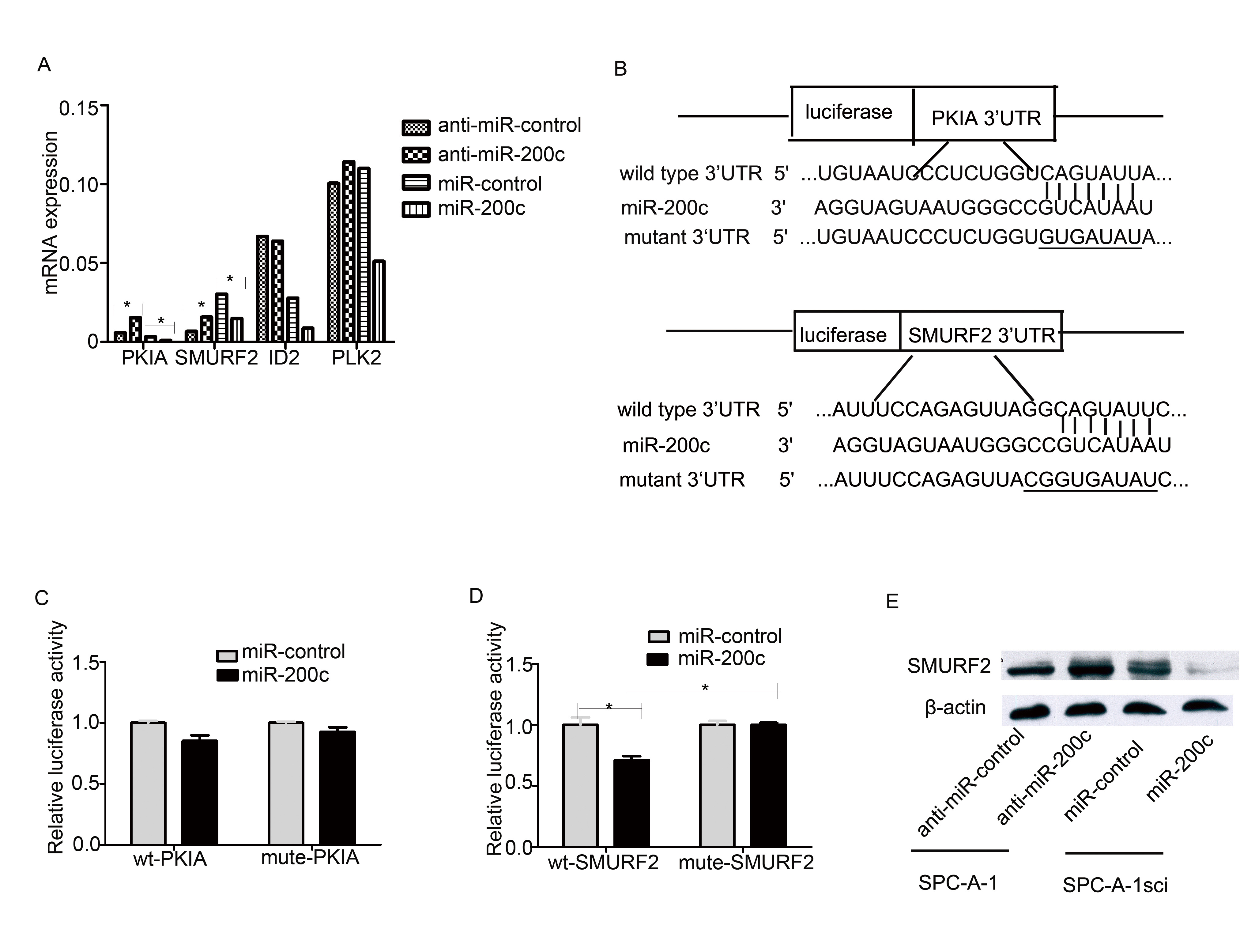

Supplement: Additional file 4: Figure S4 — Other target genes of miR-200c. (A) The ID2, SMURF2, PKIA, and PLK2 mRNA levels were determined by real-time PCR analysis after transfection with the miR-200c mimics or negative control in SPC-A-1sci cells or after transfection with the miR-200c inhibitor or negative control in SPC-A-1cells. β-actin served as an internal control. (B) The putative miR-200c binding site in the SMURF2, PKIA 3’-UTR. (C,D) Luciferase activity assays for luciferase reporters with wild-type or mutant SMURF2, PKIA 3’-UTR were performed after co-transfected with miR-200c mimics or miR-control in 293T cells. The luciferase activity of each sample was normalized to Renilla luciferase activity. (E) The SMURF2 protein levels were determined by western blot analyses after transfection with the miR-200c mimics or negative control in SPC-A-1sci cells or after transfection with the miR-200c inhibitor or negative control in SPC-A-1cells. β-actin served as an internal control. [file 1476-4598-13-166-S4.tiff]

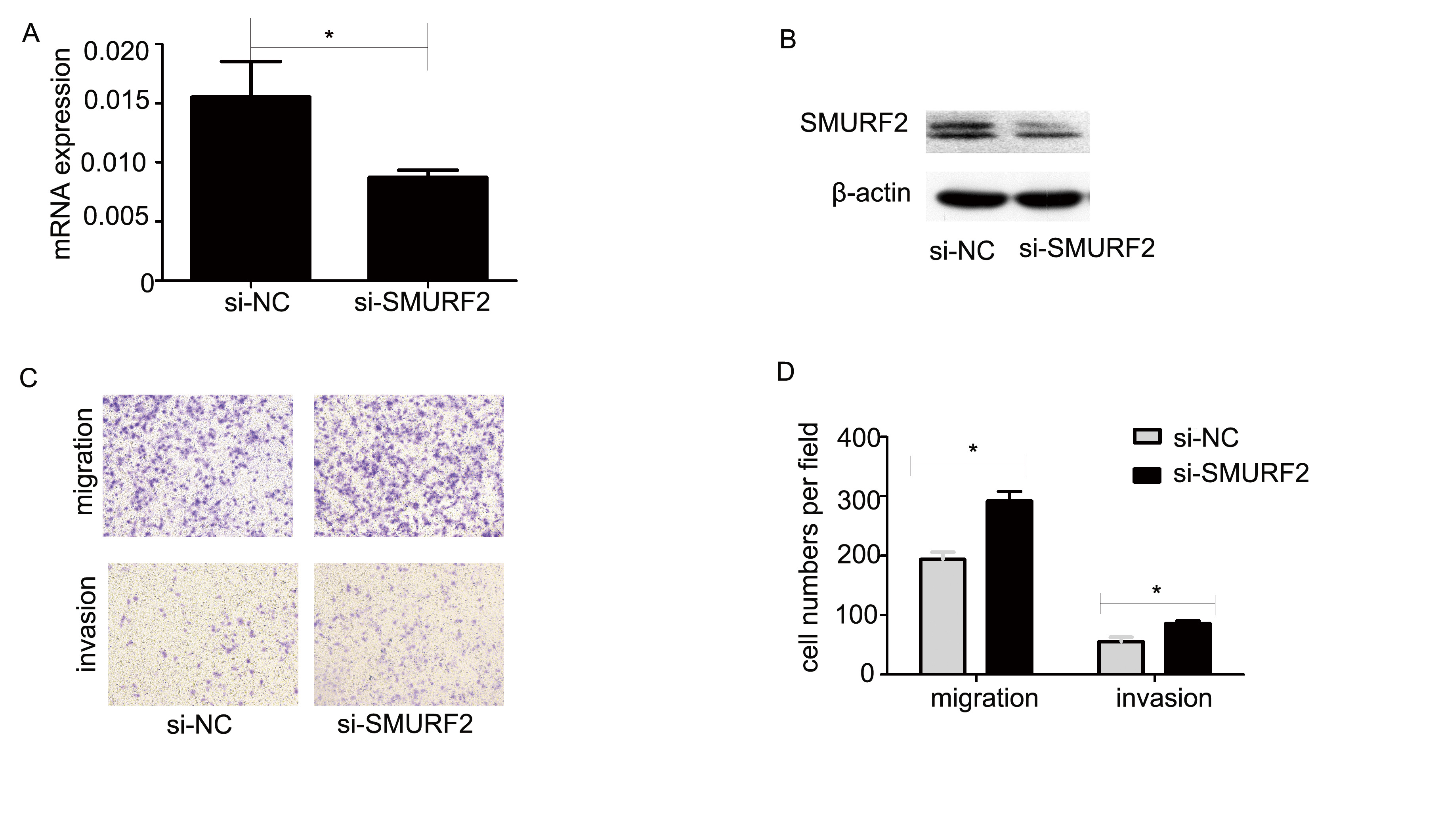

Supplement: Additional file 5: Figure S5 — Downregulating SMURF2 enhanced human NSCLC cell invasion in vitro. (A,B) SMURF2 mRNA and protein levels were determined by real-time PCR and western blot analyses after transfection with si-SMURF2 or a negative control in SPC-A-1sci cells. β-actin served as an internal control. (C,D) Transwell migration and invasion assays for SPC-A-1sci cells were performed (100x magnification) after transfection with si-SMURF2 or a negative control. The results are representative of at least three independent experiments. Statistical analysis was performed using Student’st-test. Error bars represent S.E.M. *P<0.05; **P<0.01; ***P<0.001. [file 1476-4598-13-166-S5.tiff]

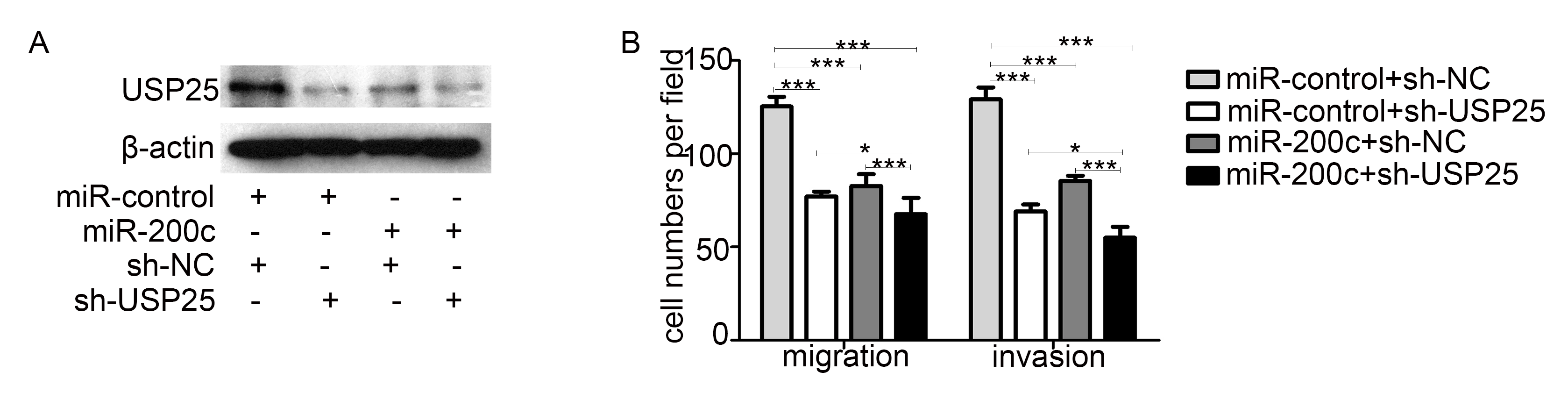

Supplement: Additional file 6: Figure S6 — Over-expression of miR-200c in USP25 knockdown SPC-A-1sci cells decreased the invasion and migration ability. (A) USP25 protein levels were determined by western blot analyses after over-expression of miR-200c in USP25 knockdown SPC-A-1sci cells. β-actin served as an internal control. (B) Transwell migration and invasion assays were performed after over-expression of miR-200c in USP25 knockdown SPC-A-1sci cells. The results are representative of at least three independent experiments. Statistical analysis was performed using Student’st-test. Error bars represent S.E.M. * P<0.05; ** P<0.01; ***P<0.001. [file 1476-4598-13-166-S6.tiff]
